# Evidence for a divergence in function between two glucocorticoid receptors from a basal teleost

**DOI:** 10.1186/1471-2148-12-137

**Published:** 2012-08-03

**Authors:** Yi Li, Armin Sturm, Phil Cunningham, Nicolas R Bury

**Affiliations:** 1 Nutritional Sciences Research Division, King’s College London, Franklin Wilkins Building, 150 Stamford Street, London SE1 9NH, UK; 2 Institute of Aquaculture, University of Stirling, Stirling, FK9 4LA, UK

## Abstract

**Background:**

Duplicated glucocorticoid receptors (GR) are present in most teleost fish. The evolutionary advantage of retaining two GRs is unclear, as no subtype specific functional traits or physiological roles have been defined. To identify factors driving the retention of duplicate GRs in teleosts, the current study examined GRs in representatives of two basal ray-finned fish taxa that emerged either side of the teleost lineage whole genome duplication event (WGD) event, the acipenseriform, *Acipenser ruthenus,* (pre-WGD) and the osteoglossimorph, *Pantodon buchholzi, (*post-WGD).

**Results:**

The study identified a single GR in *A. ruthenus* (ArGR) and two GRs in *P. buchholzi* (PbGR1 and PbGR2). Phylogenetic analyses showed that ArGR formed a distinct branch separate from the teleosts GRs. The teleost GR lineage was subdivded into two sublineages, each of which contained one of the two *P. buchholzi* GRs. ArGR, PbGR1 and PbGR2 all possess the unique 9 amino acid insert between the zinc-fingers of the DNA-binding domain that is present in one of the teleost GR lineages (GR1), but not the other (GR2). A splice variant of PbGR2 produces an isoform that lacked these 9 amino acids (PbGR2b). Cortisol stimulated transactivation activity of ArGR, PbGR2b and PbGR1 *in vitro;* with PbGR2b and PbGR1, the glucocorticoid 11-deoxycortisol was a more potent agonist than cortisol. The hormone sensitivity of PbGR2b and PbGR1 differed in the transactivation assay, with PbGR2b having lower EC50 values and greater fold induction.

**Conclusions:**

The difference in transactivation activity sensitivity between duplicated GRs of *P. buchholzi* suggests potential functional differences between the paralogs emerged early in the teleost lineage. Given the pleiotropic nature of GR function in vertebrates, this finding is in accordance with the hypothesis that duplicated GRs were potentially retained through subfunctionalisation followed by gene sharing. A 9 amino acid insert in the DNA-binding domain emerged in basal ray-finned fish GRs. However, the presence of a PbGR2 splice variant that lacks this insert, as well as the loss of the exon encoding these amino acids in the genes encoding for other teleost GR2 suggests the selection of two receptors with different DNA-binding domain structures in teleosts.

## Background

A good example of the evolution of novel traits following gene duplication is provided by the steroid nuclear receptors; a protein superfamily that includes receptors for estrogens (ER), progestins (PR), and androgens (AR), as well as the corticosteroid receptors (CRs), which in turn comprise the glucocorticoid (GR) and mineralocorticoid receptors (MR). The ancestral steroid receptor is believed to have resembled an ER [1]. ER “like” genes have been cloned from molluscs [1,2], as well as an annelid [3], and have been found to be constitutively active [2]. It is not until the urochordate amphioxus that we find an ER and the enzymes necessary for estradiol synthesis, the ligand of vertebrate ERs [4,5]. CRs are absent from amphioxus [5], and first appear in the jawless fish [6] following proposed whole genome duplication (WGD) events early in the vertebrate lineage. This ancestral CR is transcriptionally activated *in vitro* by a broad spectrum of corticosteroids, including cortisol, aldosterone, 11-deoxycorticosterone and 11-deoxycortisol [6]. A further proposed WGD event prior to the emergence of the jawed vertebrates gave rise to duplicated CRs, and thus, two CRs, a MR and a GR, are found in the Chondrichthyes [6,7] and tetrapods [8,9]. In the tetrapods the two CRs have diverged in function, with MR playing a principal role in the control of mineral balance and GR being predominantly involved in glucose metabolism and immune function [10,11]. The hormone selectivity of the mammalian MR resembles that of the ancestral CR, which is activated by a range of corticosteroids. In contrast, GR is preferentially activated by glucocorticoids [6].

There is strong evidence that another WGD event occured in the teleost lineage [12] approximately 350MYA [13] following the split of the Osteoglossidae from the Acipenseridae [14]. After the WGD event, only one MR has been retained in the teleosts [15-17]. In contrast, duplicated GRs have been found in a number of teleost species [16-20], including the most recent group to diverge between 18 to 30 MYA [13], the tetraodontiformes [18]. However, the exact advantage the retention of a duplicate GR provides is unclear because in one species, zebrafish (*Danio rerio*), there appears to have been a secondary loss of one of the GR paralogoues [21].

Following a genome duplication event the duplicated genes may take one of a number of different evolutionary trajectories. In the majority of instance (approximately 80%) mutations in the encoding region will render one of the duplicates non-functional leading to its eventual loss [12,13]. A number of theoretical models exist to explain the maintenance of duplicated genes [22]. In the gene conservation model for gene duplicate retention the paralogues retain the original function of the ancestral gene and the evolutionary advantage is to increase gene copy number [22]. On the other hand, in the duplication-degenerative-complementation (DDC) model each paralogue accumulates loss-of-function mutations and the retention of the two genes is necessary to maintain full functional capacity, a process known as sub-functionalisation [23]. The DDC model is not adaptive and other subfunctionalisation models exist [24]. Specialisation proposes that the paralogues retains the same basic function (e.g. the same substrate) as the ancestral gene, but mutations in one of the genes results in an improvement in the ancestral gene function (e.g. an increase substrate affinity) [22]. The gene sharing model accounts for situations where the ancestral gene has numerous functions. Here, mutations may be beneficial for one function of the ancestral gene but deleterious to another, and gene duplication overcomes this adaptive conflict by allowing for one of the paralogues to become specialised for one of the ancestral functions [24]. This is similar to neo-functionalisation, where one gene retains the original function, but the other alters significantly to acquire a new function (e.g. a different substrate) [22].

Glucocorticoids and their receptors have regulatory roles in many physiological processes, including carbohydrate metabolism, bone turnover, development, cell cycle, immune function, the stress response, central nervous functions, growth and reproduction [11,25]. Since the discovery of duplicated GRs in a number of teleost fish various studies have investigated the molecular characteristics, tissue expression patterns and functional traits (e.g., hormone binding or transactivation activity sensitivities) of each paralogue in an attempt to explain potential mechanisms by which the two GRs where retained [15,17-20,26-34]. However, probably because these studies have focused on the GRs of species that belong to advanced teleosts groups that emerged many millions of years after the teleost WGD event it has proven difficult to identify any unifying functional traits of each teleost GR [15,17-20,26-34]. For example, EC50 values at which hormones induce half-maximal stimulation of receptor transactivation activity reveal distinct differences in sensitivity between duplicated GRs of rainbow trout [19], but not *Astatotilapia burtoni*[15]. Consequently, to better understand the potential reason why duplicated genes were retained in the teleosts the current study set out to clone and characterise the functional traits of GRs in representatives of basal fish groups that were present prior to, and just after, the teleost WGD event: an acipenseriform, *Acipenser ruthenus*, present before the WGD and an osteoglossimorph, *Pantodon buchholzi*, that emerged after the WGD. The hypotheses being that, firstly, *P. buccholzi* possesses two GR as a consequence of the WGD and, secondly, that these *P. buchholzi* GRs either possess differing (similar to GR traits of the rainbow trout, *Oncorhychus mykiss*[19]) or similar (similar to GR traits of the *Astatotilapia burtoni*[15]) binding and transactivation activity sensitivities.

## Results

### *Pantodon buchholzi* and *Acipenser ruthenus* GR sequences data

**Figure 1:**
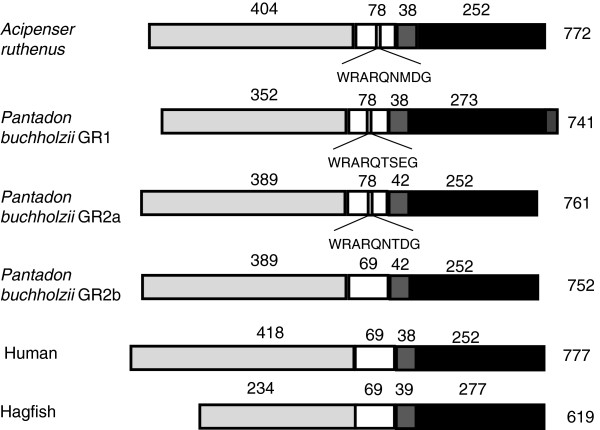
**A comparison of the A/B- domain (light gray), C-domain (white), D –domain (grey) and E-domain (black) between*Pantodon buchholzi*,*Acipenser ruthenus*, hagfish and human GRs.** The additional 9 amino acids insert between the zinc fingers of the C-domain in the *A. ruthenus* and *P. buchholzi* GR1 and 2 are indicated, and the 21 amino acid at the N-terminal *P. buchholzi* GR1 is indicated by the dark grey rectangle.

To isolate cDNA sequences encoding GRs from *Pantodon buchholzi* and *Acipenser ruthenus*, amino acid sequences of the highly conserved E-domain of a number of vertebrate GRs were aligned and degenerate primers derived (see Materials and Methods). RT-PCR produced an amplicon of the expected size with both *Pantodon buchholzi* and *Acipenser ruthenus* cDNA pooled from the livers of 3 fish. Subcloning and sequencing of PCR products revealed the presence of two GR isoforms in *P. buchholzi,* termed PbGR1 and PbGR2, which were represented at comparable numbers among analysed clones. In contrast, all analysed clones from *A. ruthenus* contained one type of sequence termed ArGR. The obtained partial cDNA sequences were extended by 5′ and 3′ RACE PCR and for each receptor, a contiguous sequence (contig) was assembled from the obtained overlapping partial sequences. The contigs contained putative complete open reading frames encoding proteins of 741 amino acids (PbGR1, [accession number, JQ791099]), 761 amino acids (PbGR2a, [JQ781069]) and 772 amino acids (ArGR, [JQ781067]) (Figure 1). The open reading frame sequence predicted by the contig assembly was confirmed by RT-PCR using total RNA from liver. The three isolated GRs show the same domain organisation as other vertebrate GRs, consisting of four domains called A/B, C, D and E. The A/B and D domains are known not to be conserved among vertebrate GRs. Accordingly, a limited degree of sequence homology was observed in these regions amongst the isolated *Pantodon buchholzi* and *Acipenser ruthenus* GRs (24-42% amino acid identity in A/B domain, 26- 50% in D domain). Despite the low degree of amino acid conservation, the A/B domains of all three basal ray-finned fish GRs possess a putative AF1 core, similar to that identified in rainbow trout [29]. The C-domain (involved in DNA binding and receptor dimerisation) and the E domain (involved in ligand binding) showed a high degree of homology among receptors (94- 97% amino acid identity in C domain, 78-83% in E domain). As with the GR1 in rainbow trout [30] and other teleosts [18], the ArGR and both PbGRs possess a 9 amino acid insertion in the linker region between the two zinc-fingers of the C domain that is lacking in hagfish and tetrapod GRs (Figure 1). However, PbGR2 was present both in a form showing the insertion, termed PbGR2a, and a variant lacking it, termed PbGR2b (JQ781068). The amino acids that make contact with the synthetic GC dexamethasone in mammalian GR [35] are conserved in ArGR and both PbGRs, which is similar to structure reported for other teleost fish GRs [19,20]. Within the E-domain there are 25 amino acids that differ between the two PbGRs. In addition, the sequence of PbGR1 has a C-terminal extension of 21 amino acids (see Additional file 1 Figure S1). 

### GR Phylogeny and ancestral sequences

**Figure 2:**
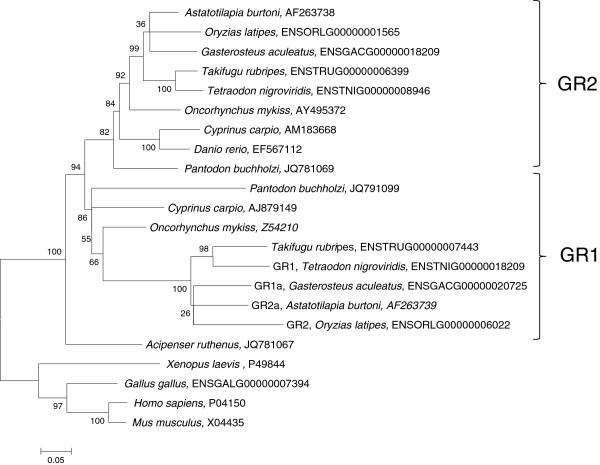
**Maximum Likelihood phylogenetic tree using full length sequence of glucocorticoid receptors.** The values represent the bootstrap values at nodes as a percent of 600 replicates as predicted by Mega 5.0 [48]. The NCBI Accession number or Ensembl Gene ID for each glucocorticoid receptor and the corresponding species name are provided.

Phylogenetic analysis of the full length GR cDNAs from a number of vertebrate species resulted in distinct, well supported lineages for vertebrate GR, teleost GR1 and teleost GR2 (Figure 2). The two PbGR subtypes grouped with two different gene lineages corresponding to subtypes 1 and 2 in the other teleosts. ArGR was basal to both teleost paralogs.

### Transactivation activity and dexamethasone binding studies

**Figure 3:**
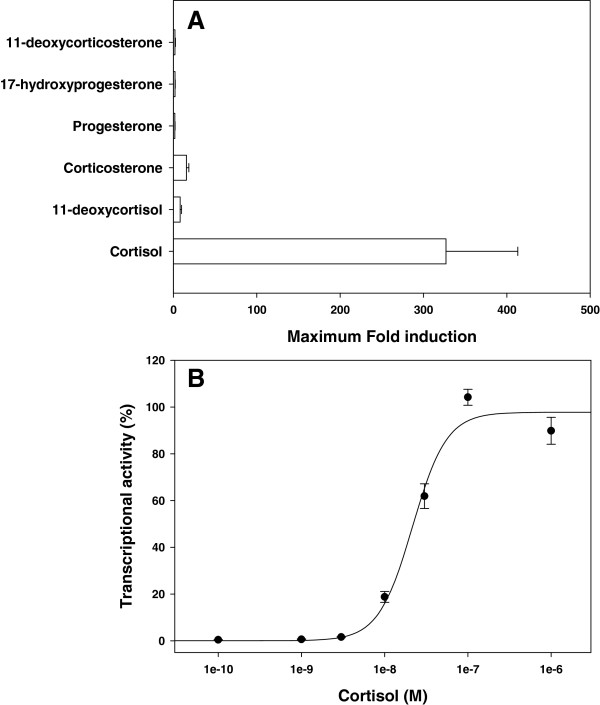
**Transactivation activity of*Acipenser ruthenus*GR.** (**A**) Maximum fold induction with a variety of glucocorticoids and mineralocorticoids above the vehicle control (n = 3 + SEM) tested over a concentration range of 10^-11^ to 10^-6^ M and dose-dependent stimulation of transactivation activity by cortisol (**B**). Values at each hormone concentration represent the average + SEM from at least 3 independent experiments. Values at each hormone concentration are normalised to maximum activity for that hormone in each experiment.

Cortisol stimulated ArGR maximum transactivation activity by 327 fold above that of the control, with an EC50 value of 21.6 ± 3.1 nM (Figure 3A and B). In contrast the other steroids tested produced only a mild induction in transactivation activity above vehicle controls, with a 15.7 and 8 fold increase for corticosterone and 11-deoxycortisol, respectively, and progesterone, 17-α hydroxyprogesterone, 11-deoxycorticosterone only producing a 1.8 to 2 fold induction (Figure 3A). Aldosterone up to 10^-6^ M was without any effect (data not shown).

**Figure 4:**
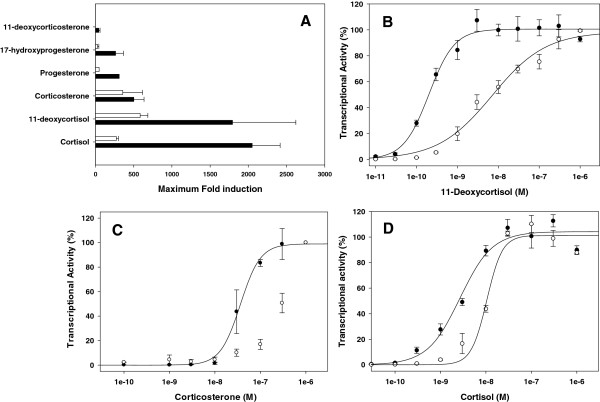
**Transactivation activity of the*Pantodon buchholzi*GR1 (PbGR1) and GR2b (PbGR2b).** (**A**) Maximum fold induction with a variety of glucocorticoids and mineralocorticoids above the vehicle control (n = 3 + SEM) tested over a concentration range of 10^-11^ to 10^-6^ M, and dose- dependent stimulation of transactivation activity by 11-deoxycortisol (**B**), corticosterone (**C**) and cortisol (**D**). Values at each hormone concentration represent the average + SEM from at least 3 independent experiments, and are normalised to maximum activity for that hormone in each experiment.

Except for 11-deoxycorticosterone, all steroids tested induced transactivation activity in PbGR1 when given at 10^-6^ M, with a hierarchy of fold induction above controls of 11-deoxycortisol, 585 fold; corticosterone, 357 fold; cortisol, 272 fold; progesterone, 48 fold; 17-α hydroxyprogesterone, 28 fold (Figure 4A). The tested steroids also stimulated PbGR2b transactivation activity at 10^-6^ M, with the hierarchy of fold induction above vehicle control being: cortisol, 2050 fold; 11-deoxycortisol, 1793 fold; corticosterone, 502 fold; progesterone, 311 fold; 17-α hydroxyprogesterone, 263 fold and 11-deoxycorticosterone, 45 fold (Figure 4A). Both PbGR1 and PbGR2b were unresponsive to aldosterone (data not shown). PbGR2a, which differs from PbGR2b by the presence of a nine-amino acid insertion into the C-domain, was not tested, as we have shown previously, by the analysis of numerous domain-swap mutants between rat GR and rainbow trout GR1 and GR2, the C-domains of which differ by the presence or absence of similar insertions, that this region of the receptor does not affect hormone sensitivity in our transactivation assay system [26,27]. Transactivation activity EC50 values could only be calculated for cortisol, 11-deoxycortisol, and corticosterone in PbGR2b, and cortisol and 11-deoxycortisol in PbGR1 (Table 1 and Figure 4 B - D). PbGR2b was more sensitive than PbGR1 with lower EC50 values in response to cortisol and 11-deoxycortisol (Table 1). This was more pronounced with 11-deoxycortisol (35.5 fold difference in EC50) than with cortisol (3.8 fold difference in EC50). 

**Table 1:** **Median effective concentrations (EC50) of glucocorticoid-dependent stimulation of*in vitro*transactivation activity by*Acipenser ruthenus*(Ar) and*Pantodon buchholzi*(Pb) GR**

	**11- deoxycortisol**	**Corticosterone**	**Cortisol**
**PbGR1**	7.1 ± 2.2	#	10.4 ± 1.4
**PbGR2b**	0.2 ± 0.02	36 ± 2.6	2.7 ± 0.6
**ArGR**	11.5 ± 4.7	#	21.6 ± 3.1

Values represent n = 3, EC50 nM ± Standard error.

# = Unable to calculate EC50, because activity did not reach a plateau at 10^-6^ M hormone concentration.

**Figure 5:**
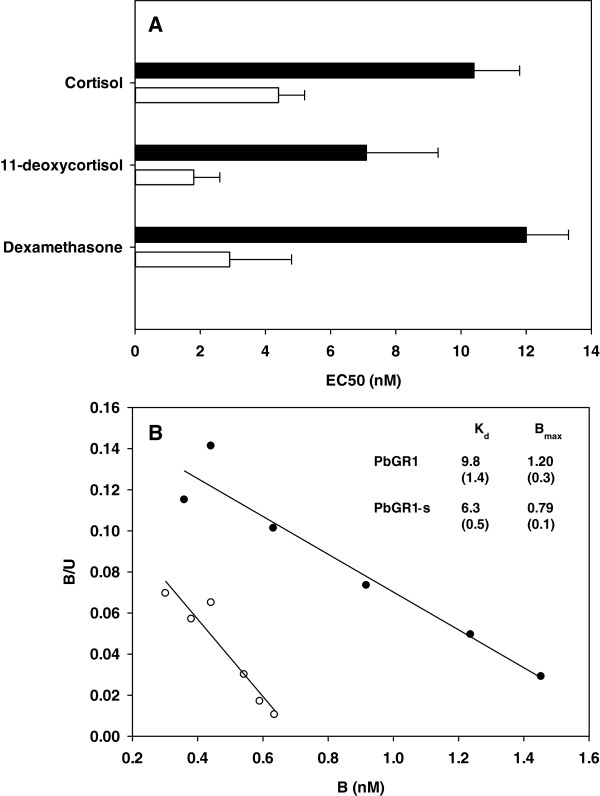
**(A) Transactivation EC50 values for*Pantodon buchholzi*GR1 (black bars) and GR1-s (white bars).** Note values for *Pantodon buchholzi* GR1 are taken form Table 1 (N = 3, average + SEM) and that GR1-s lacks the 21 amino acids at the C-terminal of GR1 (see Additional file 1 Figure S1). (**B**) Typical Scatchard plots for *Pantodon buchholzi* GR1 (black circles) and GR1-s (white circle). B, bound [^3^ H]-dexamethasone; B/U, ration of bound to unbound [^3^ H]-dexamethasone**.** The inset table provides the average binding affinities, K_d_ (nM) and maximum binding per well (nmol/well) for maximum binding per well for each receptor, values represent average (SEM) from 3 – 5 separate experiments.

Specific binding of ^3^ H-dexamethasone followed saturation kinetics in accordance with a single binding site in transiently transfected cells expressing ArGR, PbGR1 or PbGR2b, allowing the estimation of the dissociation constant *K*_d_ and the number of binding sites *B*_max_ (Table 2 and Figure 5B). PbGR2b showed a significantly greater binding affinity for ^3^ H-dexamethasone than PbGR1, as reflected by lower values of Kd, and this observation coincided with a significant difference in the EC50 and maximum fold induction values of the hormone in the transactivation assay (Table 2). There was no significant difference in *B*_max_ values between PbGR1 and PbGR2b, suggesting similar expression levels between the two receptors. The affinity of ArGR for ^3^ H-dexamethasone (*K*_d_, 11.9 ± 3.2 nM) was lower than that of PbGR1 and PbGR2b, and the number of binding sites per well was also a lot lower (Table 2). However, the ArGR was observed to have a relatively low transactivation EC50 value for dexamethasone (EC50 2.2 ± 0.7nM) compared to PbGR1 (Table 2).

**Table 2:** **Properties of*Acipenser ruthenus*(Ar) and*Pantodon buchholzi*(Pb) GRs in transactivation and binding assays with dexamethasone**

	**Transactivation EC50 (nM)**	**Maximum induction (−fold)**	**Binding K**_**d**_**(nM)**	**B**_**max**_**(nmol/well)**
**ArGR**	2.2 ± 0.7	277 ± 67	11.9 ± 3.2	0.3 ± 0.1
**PbGR1**	12.0 ± 1.3	469 ± 6	9.8 ± 1.4	1.2 ± 0.3
**PbGR2b**	1.5 ± 0.4	1079 ± 2	5.7 ± 0.7	1.17 ± 0.3

Values represent the average and SEM of 3–5 independent experiments.

An unusual C-terminal extension of 21 amino acids was found in the PbGR1 (see Additional file 1 Figure S1). Removal of this extension from PbGR1 in the mutant receptor, termed PbGR1-s, resulted in the PbGR1-s EC50 values differing from PbGR1 by 3.9 fold, 2.4 fold and 4.1 fold for 11-deoxycortisol, cortisol and dexamethasone, respectively (Figure 5A). There was also a mild shift (1.6 fold) in PbGR1-s ^3^ H-dexamethasone Kd values (Figure 5B).

## Discussion

The current study cloned and characterised the GRs from representatives of groups of fish that were present prior and emerged following the teleost-specific whole genome duplication event approximately 350 MYA [12,14]. The aim was to better understand the GR functional traits in basal ray-finned and teleost fish in order to suggest which model for gene duplication retention [22] best explains the preservation of two GRs in early teleosts. The results from our study, firstly, confirmed the presence of two GRs in a basal teleost, *P. buchholzi*, and of one GR in a basal ray-finned fish, *A. ruthenus*, which supports the hypothesis that the two teleost GRs result from the WGD in the teleost lineage [14], as has been predicted for other steroid receptors [36]. However, without a more thorough investigation of the *A. ruthenus* genome, or indeed the *P. buchholzi* genome, we cannot exclude the possibility of other GR isoforms in this species. Secondly, all GRs were transcriptionally activated in the presence of GCs, but the two *P. buchholzi* GRs showed differences in their hormone transactivation activity sensitivities indicating that this is a functional trait that differentiates the GRs in this basal teleost. A similar difference in transactivation activity sensitivities has previously been reported for the two GRs in rainbow trout [19]. Thirdly, the basal ray-finned fish and basal teleost GRs possess the unique 9 amino acid insert between the zinc fingers of the DNA –binding domain that is characteristic of one of the teleost GR sublineages, GR1 [18,30], but, is absent in the other teleost GR sublineage (GR2) [15,20] and other vertebrate GRs e.g. [8]. However, a splice variant of one of the *P. buchholzi* GRs (PbGR2) produces an isoform lacking the 9 amino acids, which suggests that there is selection for two GR isoforms with different DNA-binding domain structures in the basal teleosts.

### Hormone selectivity and sensitivity

Of the two *P. buchholzi* GRs, PbGR2b is activated in transactivation activity studies at lower hormone concentrations and with a greater maximal activity than PbGR1 (Figure 4, Tables 1 and 2). The sensitivity difference was most pronounced in the presence of 11-deoxycortisol (35.5 fold difference in EC50), and less so with cortisol (3.8 fold) and the synthetic glucocorticoid dexamethasone (8-fold). 11-deoxycortisol has been shown to induce transactivation activity of corticosteroid receptors of early vertebrates in a heterologous expression system [6], but did not stimulate the *A. ruthenus* GR. In teleosts, the effect of this hormone on GR transactivation activity has seldom been tested, but in rainbow trout it was shown to partially activate rtGR2 (10^-6^ M 11-deoxycortisol induces transactivation activity at ~20% of maximum activity induced by 10^-6^ M cortisol), but did not stimulate rtGR1 [19]. Plasma concentration of 11-deoxycortisol in teleost fish have been reported to be between 0.17 – 89 nM, depending on the species, the sex and stage in the reproductive cycle [37-39], which are similar to resting levels of circulating cortisol in teleosts [40]. However, even though these concentrations are capable of activating the *P. buchholzi* GRs (Figure 4) and 11-deoxycortisol has recently been shown to be the active glucocorticoid in the lamprey [41]; plasma concentrations in Osteoglossiforms are unknown and further work is necessary to establish whether 11-deoxycortisol acts as an active glucocorticoid *in P. buchholzi*.

To explore whether there are any significant differences between the interaction of the two *P. buchholzi* GRs and ligands that could explain the difference in transactivation activity sensitivity we modelled the docking of cortisol and 11-deoxycortisol in the *P. buchholzi* GR1 and GR2 E-domain (Additional file 1 Tables S1 and S2). Using a filter of 3.5 Å for the distance between the amino acids and the ligand the modelling exercise identified those amino acids that make up the best-fit binding pocket in the basal fish GRs. Those amino acids residues identified where the same as those predicted in the mammalian GR to interact with dexamethasone [35], Additional file 1 Figure S3. However, the model did not distinguish any useful differences between the interactions of *P. buchholzi* GR1 and GR2 with 11-deoxycortisol and cortisol (Additional file 1 Tables S1 and S2). Similarly, our attempts via point mutations to identify key amino acids responsible for difference in transactivation activity sensitivity of rainbow trout GRs proved equivocal [26]. This would suggest that the difference in transactivation activity hormone EC50 values between *P. buchholzi* GR1 and GR2b, as well as rainbow trout [26], is not a function of any easily discernable difference between the interaction of the ligand and the amino acids in the binding pocket of each receptor.

A comparison of the rainbow trout sequence with the *P. buchholzi* GR1, shows that both posses an unusual C-terminus that contains additional amino acids [PbGR1, 21 amino acids; rtGR1, 6 amino acids, Additional file 1 Figure S1]. Removal of this extension does not alter optimal hormone induction of transcriptional activity in the *P. buchholzi* GR1 mutant, but has a mild effect on the hormone EC50 values, accounting for a 3.9 fold difference in the sensitivity to 11-deoxycortisol and 2.4-fold with cortisol (Figure 5). This is analogous to rainbow trout, where the removal of C-terminal extension causes a 3.1 fold decreases in cortisol EC50 values [26]. Consequently, this unusual C-terminal region in both *P. buchholzi* and rainbow trout [26] contributes but cannot fully explain the difference in hormone sensitivity between the two GRs.

It has previously been hypothesised that the differences in GR hormone sensitivity between the teleost fish GRs paved the way for a divergence in the regulation of gene networks dependent on a broader cortisol concentration range [19,28], and the differences in glucocorticoid transactivation activity sensitivity between the two *P. buchholzi* GRs would support this hypothesis. In the current study the less sensitive *P. buchholzi* GR (PbGR1) has similar cortisol transactivation activity EC50 value to those measured in *A. ruthenus* GR, a member of a group of fish that emerged prior to the WGD event, as well as the predicted ancestral GR (AncGR2 [42]). This would suggest that the hyposensitive trait is shared between the ancestral bony vertebrate GRs and the more sensitive phenotype is derived. However, a caveat is that further studies of other basal ray-finned fish and teleosts are required to confirm this observation. Because the GR plays a role in controlling a multitude of physiological functions and development in vertebrates [11] a potential explanation for the retention of the two *P. buchholzi* GRs would be subfunctionalisation via gene sharing [24]. In this scenario one of the paralogues becomes specialised for one, or a suite, of ancestral functions and this specialisation is due to a divergence in hormone transactivation activity sensitivity between the duplicated GRs. However, this explanation for the retention of the GRs in teleost fish is not supported in other studies on more advanced teleosts were differences in hormonal sensitivity between duplicated GRs are less conspicuous, or indeed absent [15,17,20], and it is only in the rainbow trout (*Oncorhychus mykiss*) where a large difference in the hormone EC50 values between the two GRs has been measured [19,26,27]. Thus, this difference in hormone sensitivity trait between the GRs may have been selected for in early teleost and retained in other groups of fish, but it has been secondarily lost in others. Indeed, the adaptive importance of retaining two GRS can be questioned because even though 2 GRs are found in almost all of the teleost groups studied so far [15,17-20] in zebrafish (*Danio rerio*) one of the duplicated GRs has been completely lost [21].

Other models have been proposed to explain the retention of duplication GR genes. Artebery et al. [20], recently suggest that neofunctionalisation may have evolved based on difference in mineralocorticoid sensitivity of the two GRs of the plain midshipman (*Porichthys notatus*). Modelling of the GRs hormone binding pocket suggested that the exchange for Ile for Phe at position 204 (position refers to that described in [20]) would sufficiently affect the pockets hydrophobicity resulting in the altered hormone selectivity. This Ile/Phe mutation is absent in the two PbGRs, and in other species (e.g. carp (*Cyprinus carpio*) and tetradontiforms) (see Additional file 1 Figure S2). Thus, the significance of this mutation in altering receptor-hormone binding characteristics that may lead to neofunctionalisation is restricted to the plain midshipman and other species that show this mutation.

### Nine amino acid insert in the C-domain

The earliest vertebrate CR [6] and tetrapod GRs (e.g. human [8], pig [43] and rat [44]) lack an unique 9 amino acids insert between the zinc-fingers of the DNA binding domain that is present in *A. ruthenus* GR and both *P. buchholzi* GRs, indicating that this feature appeared following the split of the ray-finned fish from the lobe-finned vertebrates. The situation is, however, more complex in the basal teleosts, because a splice variant of PbGR2 that lacks these 9 amino acids is present. The significance of this observation is unclear. But, in more advanced teleosts similar splice variants of GR1 are present [30] and genome analysis reveals that the gene encoding for GR2 sublineage (Figure 2) lacks the exon present in the GR1 gene that encodes for these 9 amino acids [18]. In rainbow trout the removal of the 9 amino acid insert from the rainbow trout GR1 increases its binding strength to a single consensus glucocorticoid response element (GREs), but not to a double GRE, and this increase in binding strength translates to an increase in transcriptional activity [45]. Similarly, a single amino acid insert between the zinc-fingers of the human GR alters the transcriptional response dependent on specific response element nucleotide sequence [46]. There is a lack of information on glucocorticoid receptor recognition sites in the promoter regions of GR target genes in fish or their strength of binding to these sites. But, it is probable given the very different sequence characteristics of the DNA-binding region that the two teleost GRs will interact differently with regulatory regions of target genes.

## Conclusion

There does not appear to be a unifying explanation for why a large number of teleost fish have retained duplicated GRs. The observation that the two *P. buchholzi* GRs differ in their transactivation activity sensitivity may be a divergence in functional traits that helps explain the retention of two GRs in basal teleosts following the duplication of a GR in an ancestral teleost. However, this trait has been retained in some [19], but secondarily lost in other teleost groups [15]. Very little consideration, however, has been placed on the possibility of the divergence in the regulation of GR target genes based on alterations in the GR/promoter region interaction. The presence of an additional 9 amino acids between the zinc-fingers of the DNA domain is a unique feature of a basal ray-finned fish (*A. ruthenus*) and basal teleost GRs. But, in the basal teleost *P. buchholzi* GR2 there is a splice variant that lacks this insert. The selection for GRs with and without this 9 amino acid insert is reinforced in later teleosts where the exon encoding this region is lost in the GR2 sublineage [18]. The significance of the difference in this region for DNA recognition and target gene expression or inhibition is not fully understood, but it may be this alteration in protein structure that lead to the differential regulation of genes.

## Methods

### Cloning of the Osteoslossimorph and Acipenseriform glucocorticoid receptors

The Osteoglossimorph, *Pantodon buchholzi,* was purchased from Neil Hardy Aquatics Ltd, UK and the Acipenseriform, *Acipenser ruthenus* from Maidenhead Aquatics, UK. Fish were kept in dechlorinated Thames tap water overnight The following day the livers from three fish of undetermined sex were taken via a Home Office Schedule 1 protocol and therefor do not require a licenece and immediately frozen in liquid nitrogen and stored at −80°C until processing.

Total RNA was isolated from the livers using TRIZOL® LS Reagent (Invitrogen) and purified using RNeasy Mini Kit (Qiagen). RNA integrity was examined by electrophoresis before cDNA synthesis via SuperScript™ Reverse Transcriptase kit (Invitrogen) using random hexamers (50 ng/μl) and oligo (dT)15 primer (75 ng/μl) (Promega). Degenerate primers were designed based on alignments of the E-domain of vertebrate glucocorticoid receptors (data not shown) and those that worked for *P. buchholzi* were; 5′ TNG AYG AYC ARA TGA C 3′ and 5′- ARC ATY TCN GGR AAY TC-3′ and for *A. ruthenus*; 5′- TNG AYG AYC ARA TGA C- 3′ and 5′-ARC ATY TCN GGR AAY TC- 3′. The PCR reaction was performed using either GoTaq® Green Master Mix (Promega), at 95°C 2 min, 30–35 cycles of 94°C 30 sec, 45-65°C 45 sec, 72°C 0.5-1.5 min, followed by 72°C 7 min; or Ex Taq^TM^ (TaKaRa) 30–35 cycles of 94°C 30 sec, 45-65°C 30 sec, 72°C 0.5-1.5 min, followed by 72°C at 7 min. The resulting PCR product was run on a 1.5% agarose gel and the band of the appropriate size was excised, cleaned using Illustra GFX™ PCR DNA and Gel Band Purification Kit (GE Healthcare and Life Sciences). The purified DNA fragment was ligated into a commercial T-vector (pGEMT easy, Promega) and resulting recombinant plasmids sent away for sequencing.

To obtain full length clones, RACE was performed using SMARTer™ RACE cDNA Amplification Kit (Clontech), using specific primers designed from partial receptor cDNA sequences obtained in the previous step. RACE PCR products were subcloned as above and sequenced. With all receptors, one round of 3′ RACE and two rounds of the 5′ RACE were required to obtain the putative full open reading frame (ORF). To subclone the ORF of *A. ruthenus* GR and *P. buchholzi* GR1 and GR2 into the expression vector pcDNA3 (Invitrogen), primers were designed incorporating appropriate restriction enzyme sites (*BamH1*, Xba1) and RT-PCR was carried out using Expand High Fidelity^PLUS^ PCR System (Roche) with the following conditions 94°C for 2 min, 30–35 cycles at 94°C for 20 sec, 45-65°C for 30 sec, 72°C for 0.5 - 1.5 min, followed by 72°C 7 min extension. The resulting products purified as described above, restricted, and ligated into prepared pcDNA3 using suitable enzymes. The resulting expression vectors were termed ArGR, PbGR1, PbGR2. A further expression vector containing PbGR1 that lacked the additional 21 amino acids at the C-terminus sequence (see Additional file 1 Figure S1) was generated using a similar strategy.

### Phylogeny of the glucocorticoid receptors

Phylogenetic analysis was performed using homologous protein sequences of 18 fish GRs, an amphibian GR, an avian GR and two mammalian GRs; see Figure 2 for Accession numbers or Ensembl Gene ID. GR amino acid sequences were aligned using T-coffee [47]. Subsequently, a phylogeny was reconstructed with the Maximum Likelihood method in MEGA5, using the Jones-Taylor-Thornton substitution model and nearest-neighbour interchange [48]. Bootstrap values are reported as percentages based on 600 replicates.

### Cell culture and transactivation assays

COS-7 cells derived from African green monkey kidney and lacking endogenous expression of functional GR were maintained as described previously [19]. During and after transfection, cells were cultured in Dulbecco’s modified Eagle medium (DMEM)/nutrient mix F-12 Ham (Sigma, Poole, UK) containing 2 mM glutamine, 3.7 g/l NaHCO_3_, 100 IU/ml penicillin, 100 μg/ml streptomycin, and 2.5% denatured, dextran/charcoal-treated fetal bovine serum. The cells were grown in 24 well plates and each plate was transfected with 0.5 μg ArGR, PbGR1, PbGR2b or PbGR1-s plasmid, 10 μg firefly luciferase reporter plasmid pFC31Luc containing the murine mammary tumour virus (MMTV) promoter, 0.2 μg pBind (Promega) that expresses the *Renilla* luciferase acting as a control for transfection efficiency and 7 μg pBluescript SK + per plate using PolyFect Transfection Reagent (Qiagen). Corticosteroid treatment (progesterone, 17 α-hydroxyprogesterone, 11-deoxycorticosterone, 11-deoxycortisol, corticosterone, cortisol, aldosterone and dexamethasone, all purchased from Sigma Aldrich) started 12 h after transfection and continued for 12 hrs, after which cells were harvested using a cell lysis reagent (Promega). Firefly and *Renilla* luciferase activities were determined using the Dual Luciferase reporter assay (Promega). All experiments were performed using triplicates and repeated at least three times, except for the progesterone and aldosterone treatment where the experiment was conducted twice. For each receptor, a solvent control (ethanol) and a range of hormone concentrations 10^-11^ to 10^-6^ M were included. In further controls using empty vector instead of receptor plasmid, luciferase activities were negligible regardless of hormone treatment (data not shown). Firefly luciferase activities were divided by *Renilla* luciferase activities to correct for differences in transfection efficiency. Firefly luciferase activities of a given receptor/hormone combination were then expressed as percent of maximum activities. In the usual case, activities with the optimal glucocorticoid treatment were the highest in the data set and set to 100%. However, if a lower hormone concentration showed a higher activity, the average of this activity and all activities at higher concentrations was set to 100%. To derive median effective concentrations (EC50), a log-logistic model was fitted to the transactivation response data for each receptor using Sigmaplot version 11.0, all values are reported with standard error.

### Dexamethasone binding assays

Binding assays with ^3^ H-dexamethasone [6,7-3 H(N)] (Perkin-Elmer) were performed on COS-7 cells transiently expressing ArGR, PbGR1, PbGR1sh and PbGR2b, as previously described [26]. In brief, COS-7 cells grown in 12-well plates were transfected with receptor 5 μg plasmid containing the GR plasmid using lipofectamine 2000 (Invitrogen). 24 hrs after transfection, cells were exposed (1 hour at 37 C) to different concentrations of ^3^ H-dexamethasone [6,7-3 H(N)] (Perkin-Elmer) (1.56, 3.12, 6.25, 12.5, 25 and 50 nM) in the absence or presence of 500-fold excess unlabelled dexamethasone. After five washes with PBS, the cell were lysed using lysis buffer (Promega) and radioactivity quantified by scintillation counting. Specific binding was derived as the difference between total binding observed in cells incubated with tritiated hormone alone, and non-specific binding observed in cells exposed to tritiated hormone with an excess of unlabelled hormone. Confirming the lack of endogenous GR in COS-7 cells, total and non-specific binding were undistinguishable in non-transfected cells and in a similar range to the values of non-specific binding in GR-transfected cells (data not shown). Maximum binding (*B*_max_) and binding affinity (*K*_d_) were determined by fitting the data to Michaelis-Menten kinetics using Microsoft Microcalc version 5.0.

## Competing interests

The authors declare that they have no competing interests.

## Authors’ contribution

YL carried out the cloning and transactivation work. AS carried out the phylogenetic analysis and assisted in drafting the manuscript. PC conducted the modelling of the hormone docking in the E-domain of the glucocorticoid receptor. NB conceived the study, conducted the hormone binding studies and drafted the manuscript. All authors read the manuscript prior to submission. All authors read and approved the final manuscript.

## Supplementary Material

Additional file 1 **The supplementary data files contain an alignment of the C-terminus of the rainbow trout and*Pantodon buchholzi*GRs (Figure S1) as well as an alignment of the region of the teleost GR E-domains that possesses the isoleucine/phenylanaline mutation that is proposed by Arterbery et al. [**[20]**] to enhance mineralocorticoid selectivity in one of the duplicated GRs in the plain midshipman (Figure S2).** In addition, Table S1 and S2 provide information on the modelling of the docking of cortisol and 11-deoxycortisol in the hormone binding pocket of *Acipenser ruthenus* and *Pantodon buchholzi* GRs. Figure S3 aligns the E-domain of the *Acipenser ruthenus* and *Pantodon buchholzi* GRs and highlights those amino acids that are predicted to interact with the ligand based on our modelling exercise and matches those to the amino acids predicted to interact with dexamethasone in the mammalian GR [35]. Click here for file
