# Supplementary material for: Evidence for a divergence in function between two glucocorticoid receptors from a basal teleost
Source: BMC Evol Biol. 2012 Aug 3;12:137. doi: 10.1186/1471-2148-12-137 (PMC3457903; doi:10.1186/1471-2148-12-137)
Supplement: Additional file 1 — The supplementary data files contain an alignment of the C-terminus of the rainbow trout andPantodon buchholziGRs (Figure S1) as well as an alignment of the region of the teleost GR E-domains that possesses the isoleucine/phenylanaline mutation that is proposed by Arterbery et al. [[20]] to enhance mineralocorticoid selectivity in one of the duplicated GRs in the plain midshipman (Figure S2). In addition, Table S1 and S2 provide information on the modelling of the docking of cortisol and 11-deoxycortisol in the hormone binding pocket of Acipenser ruthenus and Pantodon buchholzi GRs. Figure S3 aligns the E-domain of the Acipenser ruthenus and Pantodon buchholzi GRs and highlights those amino acids that are predicted to interact with the ligand based on our modelling exercise and matches those to the amino acids predicted to interact with dexamethasone in the mammalian GR [35]. [file 1471-2148-12-137-S1.doc]

**Evidence for a divergence in function between two glucocorticoid receptors in a basal teleost**

Yi Li, Armin Sturm, Phil Cunningham, Nicolas.R.Bury

**Supplementary data**

**Figure S1.** ClustalW2 alignment of C terminal region of *Oncorhynchus mykiss*, rtGR1 and 2, and *Pantodon buchholzi* GR1, GR2a and GR2b.

rtGR1 PKFKDGSVKPLLFHALNHDTMP---------------

rtGR2 PKFKAGSVKPLLFHQK---------------------

PbGR1 PKFKAGSLKPLLFHQKDPAAVTDVPHHSNSVTATPNN

PbGR2b PKFKAGSVKPLLFHQK---------------------

PbGR2a PKFKAGSVKPLLFHQK---------------------

**Figure S2.** ClustalW2 alignment of duplicated GRs from teleost species that includes the regions containing the I/F mutations (in yellow) that is predicted to alter the ligand binding pocket and cause the mineralocorticoid MC sensitivity of GR2 [20]. See Figure 2 for Accession numbers or Esnembl Gene ID for these sequences and the abbreviations are as follows: *Pantodon buchholzi*, (PbGR1 and PbGR2); *Astatotilapia burtoni*, (bmGR1 and GR2b); *Cyprinus carpio*, (ccGR1 and ccGR2); *Takifugu rubripes*, (fuGR1 and 2); *Tetradon nigrividis*, (tdGr1 and 2); *Oncorhynchus mykiss*, (rtGR1 and 2); *Oryzias latipes* (mdGR1 and 2); and *Gasterosteus aculeatus* (stGR1 and 2). The *Acipenser ruthenus* (ArGR), *Myxine glutinosa* (hgGR), *Leucoraja erinacea* (SkaGR), ancestral glucocortiod receptor (ANcGR1) (GenBank: EF631197.1) and ancestral glucocorticoid receptor 2 (ANcGR2) (GenBank: EF631196.1) are provided for comparison.

rtGR1 KLLDSMQ---EMVGGLLQICFYTFVN-KSLSVEFPEMLAEIISNQLPKFKDGSVKPLLFH

rtGR2 KLLDSMHE---MVGGLLDFCFYTFVN-KSLSVEFPEMLAEIISNQLPKFKAGSVKPLLFH

stGR1 KLLDSMQ---EMVEGLLQICFYTFVN-KTLSVEFPEMLAEIISNQIPKFKDGSVKPLLFH

stGr2 KLLDSMHERPQMVGGLLSFCFYTFVN-KSLSVEFPEMLAEIISNQLPKFKAGSVKPLLFH

bmGR2b KLLDSMQ---EMVEGLLQICFYTFVN-KTLSVEFPEMLAEIISNQIPKFKDGNVKALLFH

bmGR1 KLLDSMHE---MVGGLLSFCFYTFVN-KSLSVEFPKMLAEIISNQLPKFKAGSVKPLLFH

fuGR1 ----------EMVEGLLQFCFYTFVN-KTLSIEFPEMLVEIITDQIPKFKDGSIKPLLFH

fuGR2 KLLDSMHE---MVGGLLRFCFYTFVN-KSLSVEFPEMLAEIISNQLPKFKAGSIKPLLFH

tdGR1 KLLDSMQ---EMVEGLIKFCFYTFVN-KTLSVEFPEMLVEIITNQIPKFQEGGIKALLFH

tdGR2 KLLDSMHE---MVGGLLNFCFYTFVN-KSLSVEFPEMLAEIISNQLPKFKAGSVKPLLFH

mdGR2 KLLDSMQ---EMVEGLLQICFYTFVN-KTLSVEFPDMLAEIITSQIPKFKDGGVKTLPVP

mdGR1 KLLDSMHE---MAGGLLSFCFYTFVN-KSLSVEFPEMLAEIISNQLPKFKAGSVKPLLFH

ccGR1 KLLDSMQ---EMVEGLLNFCFYTFVN-KSLSVEFPEMLAEIISHQLPKFKDGSVKPLLFH

ccGR2 KLLDSMHD---MVGGLLNFCFYTFVN-KSLSVEFPEMLAEIISNQLPKFKAGSVKSLLFH

PbGR1 KLLDFMQ---EMVGGLLNFCFYTFVN-KTLSVEFPEMLAEIISNQIPKFKAGSLKPLLFH

PbGR2b KLLDSMHD---LVGGLLNFCFYTFVN-KSLSVEFPEMLAEIISNQLPKFKAGSVKPLLFH

PbGR2a KLLDSMHD---LVGGLLNFCFYTFVN-KSLSVEFPEMLAEIISNQLPKFKAGSVKPLLFH

ArGR KLLDSMH---ELVGGLLNFCFYTFVN-KSLSVEFPEMLAEMISNQLPKFKAGSVKSLLFH

ANcGR2 KLLDSMH---EMVGGLLQFCFYTFVN-KSLSVEFPEMLAEIISNQLPKFKAGSVKPLLFH

ANcGR1 KLLDSMHD---LVGGLLQFCFYTFVQSKTLSVEFPEMLVEIISNQLPKVMAGMAKPLLFH

skaGR KLLDSMHD---LVEGLLQFCFYTFMQSKTLSVEFPEMLVEIISNQLPKVMAGMAKHLRFH

hgCR RLLDSMHN---LVGGLLEFCFMTFTQSELWSVEFPEMMSEIITAQLPHVLAGHAHALRFH

**Tables S1**: Modelling of 11- deoxycortisol docking in the E-domain of the glucorticoid receptors of *Acipenser ruthenus* and *Pantodon buchholzi* . The crystal structure of the ancestral glucocorticoid receptors interaction with dexamethasone (PBD ID: 3GN8) was used as a template to predict the structure of *Acipenser ruthenus* (ArGR) and *Pantodon buchholzi* (PbGR1 and PbGR2) glucocorticoid receptors. AutoDock [Trott O, Olson AJ: AutoDock Vina: improving the speed and accuracy of docking with a new scoring function, efficient optimization and multithreading. *J. Comput Chem* 2010, **31**: 455-461] was used to determine the binding energy and distance between key amino acids and the ligands for each receptor. Amino acid numbers correspond to those in Figure S3.

|  |  | | **11-DEOXYCORTISOL** | |  | |
| --- | --- | --- | --- | --- | --- | --- |
|  | **ArGR** | | **PbGR1** | | **PbGR2** | |
|  | Distance  (Ǻ) | Interacting  Molecule | Distance  (Ǻ) | Interacting  Molecule | Distance  (Ǻ) | Interacting  Molecule |
| 39ASN | 1.7272  1.6649  2.5886 | O2  O2  02 | 1.8643  1.7615  2.6627 | O4  O4  O4 | 1.8735  1.7707  2.6716 | O4  O4  O4 |
| 45GLN | 1.3514  1.9844 | C1  C2 | 1.7868  2.2807 | C2  C2 | 1.7766  2.2929 | C2  C2 |
| 79MET |  |  | 0.4439 | C6 | 0.4104 | C6 |
| 83LEU |  |  | 2.9276 | 02 | 2.9113 | O2 |
| 86ARG | 0.6325  1.5606  2.376 | O1  O1  O1 | 1.0751  1.9524  2.7633 | O2  O1  O1 | 1.0847  1.961  2.7751 | O1  O1  O1 |
| 98PHE | 2.2185 | O1 |  |  |  |  |
| 117GLN | 1.8441 | O3 | 2.0516 | O2 | 2.0221 | O2 |
| 207LEU |  |  | 3.1296 | C15 | 3.1572 | C15 |
| 210PHE | 1.8914  2.7598 | C15  C15 | 2.415 | C15 | 2.4623 | C15 |
| 211CYS | 3.2492  3.1398 | O4  C18 | 2.9447  2.9477 | C15  O3 | 2.9941  2.9455 | C15  O3 |
| 214THR | 0.7695  1.1821 | C18  C18 | 0.6919  1.1804 | C18  C18 | 1.2063  2.1098 | C18  C18 |
|  |  | |  | |  | |
| **Binding Energy (kcal/mol)** | **-11.5** | | **-11.5** | | **-11.5** | |

**Tables S2**: Modelling of cortisol docking in the E-domain of the glucorticoid receptors of *Acipenser ruthenus* and *Pantodon buchholzi* . The crystal structure of the ancestral glucocorticoid receptors interaction with dexamethasone (PBD ID: 3GN8) was used as a template to predict the structure of *Acipenser ruthenus* (ArGR) and *Pantodon buchholzi* (PbGR1 and PbGR2) glucocorticoid receptors. AutoDock [Trott, O. & Olson, A.J. 2010. AutoDock Vina: improving the speed and accuracy of docking with a new scoring function, efficient optimization and multithreading. *J. Comput Chem*  **31** 455-461] was used to determine the binding energy and distance between key amino acids and the ligands for each receptor. Amino acid numbers correspond to those in Figure S3.

|  |  | | **CORTISOL** | |  | |
| --- | --- | --- | --- | --- | --- | --- |
|  | **ArGR** | | **PbGR1** | | **PbGR2b** | |
|  | Distance  (Ǻ) | Interacting  Molecule | Distance  (Ǻ) | Interacting  Molecule | Distance  (Ǻ) | Interacting  Molecule |
| 39ASN | 1.7485  1.8494  2.6503 | O5  O5  O2 | 1.8733  1.7435  2.625 | O2  O2  O2 | 1.867  1.7369  2.618 | O2  O2  O2 |
| 45GLN | 1.8087  2.3294 | C2  C2 | 1.8069  2.2805 | O2  C2 | 1.8001  2.284 | O2  C2 |
| 79MET | 0.426 | C6 | 0.4461 | C6 | 0.4469 | C6 |
| 83LEU | 2.9403 | O3 | 2.9176 | O3 | 2.941 | O3 |
| 86ARG | 1.1154  1.9794  2.7804 | O1  O1  O1 | 1.0793  1.947  2.7556 | O1  O1  O1 | 1.0903  1.9509  2.7611 | O1  O1  O1 |
| 98PHE |  |  |  |  |  |  |
| 117GLN | 2.0231 | C4 | 2.0438 | O3 | 2.0303 | C4 |
| 207LEU | 3.1372 | C15 | 3.1279 | C15 | 3.1258 | C15 |
| 210PHE | 2.4443 | C15 | 2.409 | C15 | 2.399 | C15 |
| 211CYS | 2.9755  2.9374 | C15  O4 | 2.938  2.9635 | C11  O4 | 2.9265  2.9522 | C15  O4 |
| 214THR | 0.7213  1.1903 | C18  C18 | 0.6877  1.1889 | C18  C18 | 1.1733  2.0722 | C18  C18 |
|  |  |  |  | |  | |
| **Binding Energy (kcal/mol)** | **-11.8** | | **-11.8** | | **-11.7** | |

**Figure S3.** The predicted amino acids of the glucocorticoid receptor E-domain of *Acipenser ruthenus* (ArGR) and *Pantodon buchholzi* (PbGR1 and PbGR2) that interact within a distance of 3.5Å of the ligand. Those amino acids highlighted in red are those predicted to interact with 3.5 Ǻ during the current modelling exercise, and those highlighted in green and red are those amino acids identified by Bledsoe et al [35] to interact with dexamethasone.

PbGR1 PQLTPTMLSLLKAIEPEIIYAGYDSTIPDTSTRLMTTLNRLGGRQVVSAVKWAKALPGFR 60

PbGR2 PQLTPTMLSLLKAIEPETVYAGYDSTLPDTSTRLMTTLNRLGGRQVVSAVKWAKALPGFR 60

ArGR PQLTPTMLSLLEAIEPEIIYSGYDSTIPDTSTRLMSTLNRLGGRQVVAAVKWAKSLPGFR 60

***********:***** :*:*****:********:***********:******:*****

PbGR1 NLHLDDQMTLLQCSWLFLMSFGLGWRSFQQCNGSMLCFAPDLVINEERMKLPYMTDQCQQ 120

PbGR2 NLHLDGQMTLLQYSWLFLMSFGLGWRSFQQCNGTMLCFAPDLVINEERMKLPYMSDQCEQ 120

ArGR SLHLDDQMTLLQCSWLFLMSFSLGWRSYKQSNGSMLCFAPDLVINDERMKLPYMFEQCEQ 120

.****.****** ********.***** ::*.**:***********:******** :**:*

PbGR1 MLKISNEFVRLQVTYEEYLCMKVLLLLSTVPKDGLKSQAIFDELRMSYIKELGKAIAKRE 180

PbGR2 MMKIANEFVRLQISHDEYLCMKVLLLLSTVPKDGLKSQAVFDEIRMSYIKELGKAIVKRE 180

ArGR MLKISNELVRLQLSYDEYLCMKVLLLLSSVPKEGLKSQGVFDEIRMTYIKELGKAIVKRE 180

*:**:**:****::::************:***:*****.:***:**:*********.***

PbGR1 ENTSQNWQRFYQLTKLLDFMQEMVGGLLNFCFYTFVNKTLSVEFPEMLAEIISNQIPKFK 240

PbGR2 ENSSQNWQRFYQLTKLLDSMHDLVGGLLNFCFYTFVNKSLSVEFPEMLAEIISNQLPKFK 240

ArGR ENSSQNWQRFYQLTKLLDSMHELVGGLLNFCFYTFVNKSLSVEFPEMLAEMISNQLPKFK 240

**:*************** *:::***************:***********:****:****

PbGR1 AGSLKPLLFHQKDPAAVTDVPHHSNSVTATPNN 273

PbGR2 AGSVKPLLFHQK--------------------- 252

ArGR AGSVKSLLFHQK--------------------- 252

***:*.******
